# Lysosomes and LAMPs as Autophagy Drivers of Drug Resistance in Colorectal Cancer

**DOI:** 10.3390/cells14080574

**Published:** 2025-04-11

**Authors:** Tsvetomira Ivanova, Yordan Sbirkov, Maria Kazakova, Victoria Sarafian

**Affiliations:** 1 Department of Medical Biology, Medical University-Plovdiv, 4000 Plovdiv, Bulgaria; yordan.sbirkov@mu-plovdiv.bg (Y.S.); mariya.kazakova@mu-plovdiv.bg (M.K.); 2 Research Division of Molecular and Regenerative Medicine, Research Institute at Medical University-Plovdiv, 4000 Plovdiv, Bulgaria

**Keywords:** LAMPs, lysosomes, autophagy, drug resistance, CRC

## Abstract

Colorectal cancer (CRC) is among the most malignant pathologies worldwide. A major factor contributing to the poor prognosis of neoplastic diseases is the development of drug resistance. It significantly reduces the utility of most therapeutic protocols and necessitates the search for novel biomarkers and treatment strategies to combat cancer. An evolutionarily conserved catabolic mechanism, autophagy maintains nutrient recycling and metabolic adaptation and is also closely related to carcinogenesis, playing a dual role. Autophagy inhibition can limit the growth of tumors and improve the response to cancer therapeutics. Lysosomes, key players in autophagy, are also considered promising targets for anticancer treatment. There are still insufficient data on the role of poorly studied glycoproteins related to autophagy, such as the lysosome-associated membrane glycoproteins (LAMPs). They can act as multifunctional molecules involved in a multitude of processes like autophagy and cancer development. In the current review, we summarize the recent data on the double-faceted role of autophagy in cancer with a focus on drug resistance in CRC and on the roles of lysosomes and LAMPs in these interconnected processes. Several lysosomotropic drugs are discussed as options to overcome cancer cell chemoresistance. The complex networks that underline defined autophagic pathways in the context of CRC carcinogenesis and the role of autophagy, especially of LAMPs as drivers of drug resistance, are outlined.

## 1. Introduction

The burden of cancer incidence and mortality is rapidly growing worldwide, with colorectal cancer (CRC) ranked third amongst the most common neoplasms [1]. Despite the improvement in response rates with various therapeutic strategies such as monoclonal antibodies combined with chemotherapy, immunotherapy, and checkpoint inhibitors, the five-year survival rate for metastatic CRC is only about 12% [2]. A major obstacle to successful therapy outcomes is the development of drug resistance, which is the most critical factor in the poor prognosis of neoplastic diseases, including CRC [3]. Due to extensive multidisciplinary research, a variety of drug resistance pathways have been unraveled, with autophagy being one of the most intricate phenomena.

An evolutionarily conserved catabolic mechanism, autophagy maintains nutrient recycling and metabolic adaptation. Several studies in recent years have shown the link between carcinogenesis and autophagy. In cancer, autophagy acts as a double-edged sword. It inhibits the initial steps of tumor formation by activating cell death, while in advanced stages, it promotes disease progression. The complex pathophysiological role of autophagy is reflected by its ability to serve as either a pro-survival or a pro-death driver. In some cases, autophagy confers chemoresistance and facilitates cell survival, whereas in others it inhibits tumor initiation by preventing cell damage or fosters chemosensitivity by triggering cell death [4,5]. Activated autophagy supports tumor growth by delivering nutrients, maintaining metabolic homeostasis, and decreasing the expression of p53 and MHC class I molecules in cancer cells [6]. Importantly, cytoprotective autophagy may be triggered by chemotherapy, targeted therapy, and radiotherapy, and thus it can protect cancer cells by providing recycled nutrients and energy to maintain their survival [7]. In addition, it has been shown that during malignant transformation, a reprogramming of the autophagy process can take place, resulting in the inactivation of tumor suppressor genes and proteins and the concomitant activation of oncogenes [8].

The regulation of autophagy is intertwined with the dynamics of the lysosomal system, corroborating the crucial function of lysosomes in this process. These membrane-bound cell organelles, similarly to autophagy, may play a dual role in cancer progression [9]. They are able to affect invasive tumor growth and vascular development, as well as to protect tumor cells from chemotherapeutics, thus contributing to the development of drug resistance [10].

Changes in autophagy networks and lysosomal functions are therefore implicated not only in cancer pathogenesis but also in potential therapeutic approaches [11]. Autophagy inhibition can limit the growth of tumors and improve the response to cancer therapeutics [12,13,14,15]. Therefore, targeting autophagy is an attractive treatment strategy for a wide range of cancers [16,17,18,19].

Lysosomal cell death may also play an important role in antitumor therapy. The concept of lysosomotropic agents launched by de Duve et al. [20] was further developed by focusing on their activity as autophagy inducers with possible applications in a variety of pathologies, including cancer [21].

Drug resistance significantly reduces the utility of most therapeutic protocols and necessitates the search for novel biomarkers and treatment strategies to combat cancer.

In this respect, there are still insufficient data on the role of poorly studied glycoproteins related to autophagy, such as the lysosome-associated membrane glycoproteins (LAMPs). It has been proposed that LAMPs’ complete functions are far beyond their initially suggested roles in maintaining the structural integrity of the lysosomal compartment [18]. They seem to act as multifunctional proteins involved in a multitude of processes like autophagy and cancer development.

The aim of the present review was to summarize and align the double-faceted role of autophagy in cancer with a focus on drug resistance in CRC and on the implication of lysosomes and LAMPs in these interconnected processes.

## 2. Autophagy, Lysosomes, and LAMPs—Basic Concepts

Autophagy is an important catabolic process that upkeeps the well-being of eukaryotic cells by degrading damaged or redundant cell components, such as proteins and organelles. Thus, it maintains cellular homeostasis either by providing energy sources, building material for the de novo synthesis of new macromolecules, or by eliminating photogenic agents. Autophagy can be induced by various environmental conditions like starvation, growth factor depletion, hormonal and cytokine levels, or different pathogens. Lysosomes have an indispensable role in autophagy, as not only do all paths lead to them, but they are also involved in the activation of the “self-eating” process.

Since their discovery by de Duve in 1955, lysosomes have been ubiquitously detected in most animal cells. Lysosomes are spherical organelles that digest and eliminate cellular waste of extracellular origin via endocytosis or that of intracellular origin through autophagy. Similarly, they can also destroy bacteria and viruses engulfed by phagocytosis [22]. As functional cellular structures, lysosomes must be maintained intact, which is achieved mainly by a few membrane proteins, the lysosome-associated membrane proteins (LAMP-1, LAMP-2), and Hsp70. Notably, an alteration in the permeabilization of the lysosome membrane can signal cell death, either by apoptosis or necrosis. The pathway through which the cell will die is determined by the level of damage and leakage from the lysosomes [23]. Depending on the nature of the cargo and the mode of delivery to lysosomes, either in a selective or non-selective manner, different types of autophagy are activated. Thus, for example, we can have mitophagy, reticulophagy, xenophagy, ferritinophagy based on the cargo, or ubiquitin-dependent or -independent autophagy with respect to the selective mode of delivery to the lysosomes [24].

There are three major types of autophagy—microautophagy, chaperone-mediated autophagy (CMA), and macroautophagy—and different LAMP molecules are implicated in all. Microautophagy takes place directly in the lysosome, where non-selective uptake and degradation of cytosolic material by invagination of the lysosomal membrane occur. The direct delivery of nucleic acids into lysosomes involves RNA/DNA autophagy, where LAMP-2C binds and translocates the nucleic acid to the lumen of the lysosome [24]. CMA is a process that also occurs directly on the membrane of the lysosome, but in a selective manner. It involves the chaperone Hsp70, which recognizes and binds proteins with a specific amino acid motif. Once recognized, these substrates are translocated by the LAMP-2A isoform directly onto the lysosomal membrane for subsequent degradation [25,26]. Some reports suggest a link between CMA and resistance to antitumor therapy [14,27]. In various cancer cell types, a fold increase in CMA is recorded, indicating that CMA promotes cancer cells’ growth and development. Macroautophagy, the most well-studied form of autophagy, entails the selective removal of unwanted, spare, or damaged organelles such as mitochondria (mitophagy), peroxisomes (pexophagy), lipid droplets (lipophagy), and ER (reticulophagy). This process involves the fusion of the lysosome with the autophagosome. Macroautophagy is initiated either by an mTOR-dependent mechanism, where mTORC1, one of the main complexes that functions as a nutrient/energy/redox sensor and controls protein synthesis, dissociates from the assembled ULK protein complex (ULK1, FIP200, Atg13, Atg101). Alternatively, the mTOR-independent pathway can be induced by altered transcription of macroautophagy genes and impaired inositol levels. This in turn leads to the formation of an autophagosome, a double-membrane structure that selectively engulfs targeted molecules. The autophagosome and the lysosome then combine, forming an autolysosome, where the sequestered content is degraded, and the digested products are released in the cytosol. Beclin1, LC3B, and LAMP-1 are among the key protein components playing crucial roles in different stages of macroautophagy. Beclin1 promotes autophagosome formation, LC3B is an integral constituent of the autophagosome membrane, and LAMP-1 is a primary constituent of the lysosomal membrane [28].

There are about 40 currently known autophagy-related genes (ATGs) responsible for the core machinery of autophagosomes and the selective modes of autophagy. The fact that these genes have been conserved through the evolution of eukaryotic cells indicates the fundamental importance of the process. Numerous studies have demonstrated a link between autophagy and tumorigenesis, focusing on the interactions between autophagy, targeted therapy, and drug resistance. However, it is not well understood if different targeted therapies will elicit similar effects on autophagy with respect to promoting drug resistance [29]. Lastly, even though cells can die through at least 10 different mechanisms [30], autophagic cell death is one of the three distinct types of cell death, together with apoptosis and necrosis [31]. Of note, while an increase in autophagosomes is often seen in dying cells, cell death can also occur with concomitant autophagy rather than being induced by autophagy itself [32]. Therefore, it is now well established that autophagy is not only involved in cellular homeostasis through digestion and recycling of abnormal or accumulated cytosolic components but is also a key element in tumorigenesis [14,27].

## 3. Autophagy in Colorectal Cancer and in the Tumor Microenvironment

Colorectal cancer (CRC) is the third-leading malignant pathology in the world. Unfortunately, the rate of occurrence is increasing drastically among young adults [33,34]. Improper lifestyle, obesity, gene mutations, chronic inflammatory intestinal processes, and genetic predisposition are some of the causes of this multifactorial disease [35]. The poor prognosis is due not only to the high percentage of late-diagnosed cases but also to the resistance acquired by tumor cells to chemo- and radiotherapy [36]. This fact, combined with tumor heterogeneity, drives the search for a new, complex approach for earlier and more personalized CRC therapy.

The context-dependent role of autophagy can be clearly illustrated with several examples with opposite outcomes from one and the same signaling axis—the mTOR pathway [37]. First, grape-seed procyanidin B2 (PB2) and justicidin A can both induce autophagy and apoptosis through activation of PI3/AKT [38,39]. Oridonin achieves the same through triggering the AMPK/mTOR pathway [40]. The concept of autophagy-induced apoptosis in CRC was demonstrated by a study with itraconazole that inhibited Hedgehog signaling and reduced tumor growth in xenografts with HCT-116 cells (but not SW-480 cells) [41].

On the other hand, autophagy can counteract apoptosis too. Therefore, inhibition of autophagy can in fact enhance the effect of a number of antitumor compounds that activate autophagy through inhibition of mTOR signaling: toxicarioside O (TCO) [42], extract from *Antrodia salmonea* (AS) [43], lomerizine 2HCl [44], salidroside [45], myricetin [46], and many others.

In the early stages of tumor development, autophagy mostly plays a protective role by suppressing tumor growth and thus preventing the accumulation of toxic waste. In this regard, increased Beclin1 expression is associated with favorable prognosis of CRC patients [47]. In another CRC patient study, Zhang et al. showed that LC3 is suppressed along with a reduction in the expression of Beclin1. In vitro experiments revealed that overexpression of Beclin1 inhibits CRC cell growth and enhances the antitumor potency of rapamycin [48]. Interestingly low LC3B, Beclin1, and ATG5 levels are associated with poor patient survival in CRC [49]. In addition, FAM134B, a reticulophagy receptor, was shown to act as a cancer repressor in colon cancer, as lower levels or knockdown promote tumor growth [50]. Furthermore, the same group demonstrated that a decrease in FAM134B affects autophagy through the WNT/β-catenin pathway, thus promoting colon tumorigenesis [51].

Furthermore, Zheng et al. linked enhanced LC3 autophagy with colon cancer aggressiveness [52]. In another study, the researchers found an association of CRC tumor aggressiveness and p53 mutation status with induced LC3B level [53]. High Beclin1 expression was also linked with hypoxia and tumor progression in a different CRC-examined group [54]. Additionally, it has been found that ATG7 levels are critical for CRC cell viability. Tumor invasion and lymph node metastasis are also related to increased ATG5 and ATG10 protein amounts [55].

**Figure 1:**
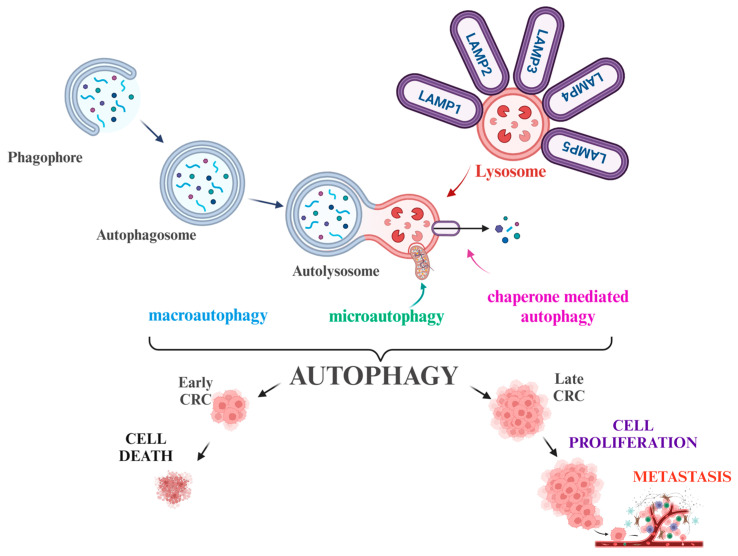
Autophagy, lysosomes, and LAMPs in CRC. The figure schematically represents the double-edged role of autophagy in tumorigenesis. In the early stages of tumor development, autophagy mostly plays a protective role by suppressing tumor growth and thus preventing the accumulation of toxic waste, while in advanced stages, it promotes tumor proliferation and disease progression. The complex pathophysiological role of autophagy is reflected by its ability to serve as either a pro-survival or a pro-death driver.

In summary, as a housekeeping process to avoid carcinogenesis and to reduce tumor development at earlier steps, autophagy exerts a tumor-suppressing role (Figure 1).

However, in advanced stages, it can act as a survival mechanism for cancer cells by increasing their tolerance to nutrients and oxygen deprivation within the tumor microenvironment (TME) or by inducing metabolic changes and treatment resistance (Figure 1). In a majority of studies, however, augmented autophagy refers to poor prognosis and metastasis (Figure 1).

The opposing effects of autophagy have been associated with a wide range of cancer-related processes. There are numerous examples of the importance of autophagy for survival of cancer cells during therapy. Importantly, the benefits of inhibiting this process are strongly supported by both in vitro and in vivo experiments. For example, the clinically approved tyrosine kinase inhibitor apatinib was shown to induce endoplasmic reticulum (ER) stress and to activate autophagy. If, however, chloroquine is used together with apatinib, this enhances apoptosis in vitro and reduces tumor volume in xenografts [56]. Similarly, inhibition of autophagy by overexpression of miR-22 (which downregulates LC3) and chloroquine can enhance the effect of 5-FU in cancer cell lines and xenografts [57,58].

The importance of tumor heterogeneity can be highlighted by a study showing that autophagy is activated in CRC stem cells (CD133-positive cells) upon photodynamic therapy-induced apoptosis. The protective role of autophagy in this case was demonstrated by silencing of *ATG5* and the use of chloroquine (CQ) and 3-methyladenine (3-MA). Both strategies successfully enhanced apoptosis in CRC stem cells, but not in CD133-negative cells. Decreased colonosphere formation in vitro and decreased tumor incidence and volume in xenograft mouse models was reported [59].

A similar relationship was shown between the natural plant product toxicarioside O (TCO) and chloroquine. TCO induced apoptosis, but also autophagy through SIRT1 upregulation and Akt/mTOR inhibition as a rescue mechanism, yet blocking autophagy enhanced the cytotoxic effects of TCO in CRC cell lines [42]. In accordance with these findings, Yang et al. investigated the effects of the medicinal fungus *Antrodia salmonea* (AS) extracts, both in vitro and in vivo. The authors again observed augmented cryoprotective autophagy after treatment with AS through the inhibition of mTOR signaling, but also described ERK activation as an important step in this rescue mechanism of CRC cells [43].

Of note, CRC cells do not modulate autophagy only in response to chemotherapeutics. Perhaps the first events that alter autophagy are changes in the TME—hypoxia and nutrient deprivation, cancer-associated fibroblasts, and the gut microbiota.

Most well-examined TME factors that affect autophagy in CRC cells are hypoxia and nutrient deprivation, where this process is largely hijacked as a rescue mechanism. For example, glutamine deprivation and asparagine depletion (and glutamine deamination) lead to growth arrest and apoptosis of CRC cell lines with concomitant activation of autophagy. Targeting this pro-survival process with chloroquine, however, can enhance the anticancer effect of inhibition of glutaminolysis [60]. Interestingly, under hypoxia, proline oxidase (POX) can be upregulated in an HIF1-independent manner, which can result in the generation of reactive oxygen species (ROS) and activation of pro-survival autophagy [61]. Of note, hypoxia has been shown to trigger autophagy in tumor-initiating CRC cells through the phosphorylation of ezrin (and likely activation of PRKC/PKC signaling) [62].

Besides hypoxia and nutrient deprivation, another TME factor is cancer-associated fibroblasts (CAFs). The interactions between stromal and CRC cells in the context of autophagy have been suggested by several studies showing that tumors induce this process in their surrounding cells so that they provide nutrients and ensure cancer cell growth and even metastasis [63]. However, CAFs can also activate autophagy in CRC cells, and one way is by releasing the extracellular matrix protein periostin. It binds to integrin receptors (ITGα5β1 or α6β4), which can in turn trigger the AKT pathway. Thus, autophagy is suppressed, enhancing the migration of CRC cells in 3D cultures. Importantly, Thongchot et al. also found in 410 tumor tissue samples that high periostin levels detected by immunohistochemistry correlated with lymph node and distant metastases as well as with poorer 3- and 5-year overall survival [64].

Lastly, a critical component of the TME of CRC in particular is the gut microbiota. With regard to the interplay between bacteria and autophagy, Yu et al. provided details from in silico, ex vivo, and in vitro experiments. The authors demonstrated that *Fusobacterium nucleatum* can interact with TLR4 receptors on HCT116 and HT29 CRC cells and activate MYD88 signaling, which in turn upregulate autophagy. Notably, knockdown of these two molecules decreases chemoresistance to oxaliplatin in xenografts. The clinical relevance of the *F. nucleatum*–TLR4–MYD88–autophagy (ULK1 and ATG7)–drug resistance axis has been demonstrated through correlation analysis showing that patients harboring the bacteria have higher expression of autophagy markers and higher CRC recurrence [65].

## 4. LAMPs

The latest research has unveiled some remarkable insights into the possible role of LAMPs as autophagy-related molecules in CRC drug resistance [66,67]. These findings hold immense potential to revolutionize cancer treatment and improve patient outcomes, presenting a promising avenue for researchers and clinicians alike.

There are five known lysosome-associated membrane proteins identified so far LAMP-1, LAMP2, LAMP3, CD68/macrosialin/LAMP-4, and BAD-LAMP/LAMP-5. Interestingly, a role in cancer is suggested for all five molecules (Table 1).

LAMP-1 and LAMP-2, the most ubiquitous, are implicated in a multitude of cellular processes, including phagocytosis, lipid transport, and aging, although their functions are not fully understood [68]. LAMP-1 is routinely used as a lysosome marker, and LAMP-1-positive organelles are often referred to as lysosomal compartments [69]. Predominantly, LAMP-1 is involved in macroautophagy, the process when the autophagosome is created. Following delivery to the vacuole or lysosome, the cargo is degraded, and the macromolecules are released into the cytosol for reuse [28]. The LAMP-2 gene produces three alternative splicing variants—LAMP-2A, LAMP-2B, and LAMP-2C. LAMP-2A is a key component in chaperone-mediated autophagy (CMA) [70]. An alteration of this pathway has been demonstrated in a variety of pathological conditions, such as lysosomal storage diseases, cancer, and neurodegeneration [71,72].

Like LAMP-2B, LAMP-2A is also constitutively presented in most tissues and cells, especially in cardiac and skeletal muscle, and brain [73]. It is suggested that the proteins, as part of the exosome membrane, are involved in cardiovascular diseases and cancer [74]. LAMP-2C has a more restricted tissue distribution, as it is exclusively expressed on mature dendritic cells [75]. LAMP-2C serves as a receptor for nucleic acid autophagy, in which DNA and RNA are engulfed directly into lysosomes for degradation. LAMP-4 (CD68) is poorly studied, but its preferential location within late endosomes may suggest a role in antigen processing [76]. It is used as a histo- or cytochemical marker of inflammation related to monocytes/macrophages [77]. So far, the available information on CD68 expression in normal or pathological conditions is insufficient. LAMP-5, also known as brain and dendritic cell-associated LAMP (BAD-LAMP), plays a role in short-term synaptic plasticity. Although LAMP-5 belongs to the LAMP family based on structural similarity in the LAMP domain, it is different from the other related proteins in terms of tissue-specific expression [78]. As LAMP-5 is found in cortical pyramidal neurons, mainly in distinct intracellular vesicles, it is expected to play a different role from that of LAMP-1 and LAMP-2. It could likely contribute to distinct functions in the central nervous system [79].

## 5. LAMPs in Cancer and in CRC

Resistance to chemotherapeutic agents is a major problem in oncology, which limits the effectiveness of the applied therapy [80]. The implication of autophagy in regulating cancer cell death or survival is still controversial. LAMPs are thought to play a key role in the progression of many types of cancer by facilitating neoangiogenesis and tissue remodeling [81]. Moreover, several cancer cell lines have demonstrated an increased expression of LAMPs on their plasma membranes. This finding, coupled with their communication with extracellular matrix proteins, may suggest involvement in tumor progression [82].

The overexpression of LAMP-1 in epithelial ovarian cancer is significantly associated with metastasis and unfavorable patient survival [83]. A similar trend is observed in breast cancer [84] and BCL [85].

Increased LAMP-2A expression has been determined in several types of cancer, including breast cancer [86], CRC [26,87], liver cancer [13], and stomach cancer [88]. High LAMP-2A expression has been detected in breast cancer, gastric cancer cells, and non-small-cell lung cancer [88]. While LAMP-2A has been proven to be implicated in the promotion of tumor growth, LAMP-2B and LAMP-2C are rarely reported in the field of cancer [89,90]. Knockdown of LAMP-2B destroys the fusion of autophagosomes with lysosomes and impairs resveratrol-induced apoptosis in human CRC cells [74].

The expression of LAMP-3 in human ovarian cancer is correlated with a poor clinical outcome and metastasis [91]. Further, loss of LAMP-3 markedly inhibited the motility and metastasis of esophageal cancer cells [92]. Recently, the interaction between LAMP-3 and the ribosomal protein L21 was proven to lead to the formation of focal adhesions. This evidence of the prometastatic effect of RPL21 and LAMP-3 highlights their role as therapeutic targets in cases of CRC metastases [93]. An increased expression of LAMP-3 across multiple cancers indicates its potential as a guiding marker for pathological staging and immune infiltration [94].

Interestingly, LAMP-4 (CD68) is associated with tumor immunity in the TME, as LAMP-4 levels are positively related to immune cell infiltration, such as dendritic cells, macrophages, monocytes, and neutrophils. Various small molecules against LAMP-4 are used to efficiently suppress the growth of cancer cell lines [95]. LAMP-4/CD68 has been proposed as a cancer-associated diagnostic and prognostic marker [96]. Its expression by tumor cells is not occasional, since metastatic tumor cells express immune markers to escape macrophage-mediated phagocytosis [97]. Even though CD68 expression is considered a valuable tool for the assessment of tumor grade and metastatic potential, its contribution to CRC development and drug resistance remains unknown [98].

Elevated LAMP-5 has been reported in various types of cancer. High expression of LAMP-5 is associated with a poor prognosis in mixed-lineage leukemia, while knocking down LAMP-5 improves the survival rate in vivo [98]. LAMP-5 is a prognostic biomarker in both gastric cancer and CRC [99]. Additionally, an association between LAMP-5 and cancer progression through autophagy and macrophage invasion has also been reported [99]. Thus, in metastatic tissues, LAMP-5 expression is increased, while knocking out LAMP-5 significantly inhibits the proliferation, invasion, and migration of gastric cancer cells by promoting apoptosis, cell cycle arrest, and cancer stemness [100]. It has been reported that higher mutational burden of LAMP-5 in combination with other genes is associated with prognosis and abundance of tumor-infiltrating lymphocytes in colon adenocarcinoma [101]. Moreover, LAMP-5 promotes metastatic potential, as high LAMP-5 mRNA levels are significantly associated with a worse prognosis in gastric cancer cells [100].

Table 1 summarizes LAMPs’ expression and involvement in human CRC.

**Table 1:** An overview of LAMPs’ involvement in human CRC.

LAMP Protein	Sample/Cell-Based	Expression in Cancer	Role
LAMP-1 LAMP-2	mCRC, screening of clinical material	Increased amount of poly-N-acetyllactosamine in both LAMPs of highly metastatic cells	Associated with tumor progression and acquisition of a metastatic phenotype; Inhibition of poly-N-acetyllactosamine synthesis apparently reduces tumorigenicity [102].
LAMP-1 LAMP-2	CRC, screening of clinical material	Highly expressed in the epithelium of CRC compared to normal mucosa. Increased expression of LAMP-1 and LAMP-2A, and LAMP-2B by NB	LAMPs are related to neoplastic progression [89].
LAMP-1	Caco2 cells, in vitro cell-based studies	Higher proportion of olylactosaminoglycans on LAMP-1 in undifferentiated CaCo2 cells	LAMP-1 of CaCo2 cells contains polylactosaminoglycans, that undergo changes in glycosylation with differentiation [103].
LAMP-1 LAMP-2	CRC, screening of clinical material	Upregulation of TSC403 transcript sharing a similar amino acid sequences with LAMP-1 and LAMP-2	Related to the development and/or progression of cancer in humans [104].
LAMP-2	CRC, screening of clinical material	Upregulated LAMP-2 levels are stage dependent	Hypomethylation of LAMP-2 associated with upregulated gene levels. In silico analysis of miRNAs targeting LAMP-2 levels in CRC [87].
LAMP-2A	GC, screening of clinical material; BGC823 and AGS cell lines, in vitro cell-based studies	Upregulated LAMP-2A	LAMP-2A and CMA target RND3 for constant degradation to sustain the rapid proliferation of gastric colon cells [88].
LAMP-2A	CRC, screening of clinical material; CT26 cells, in vitro cell-based studies; BALB/c mice	Upregulated LAMP-2A	Inhibition of CMA in CT26 cells facilitates apoptosis [105].
LAMP-2A	CRC, screening of clinical material; HCT116, HCT8, SW480, in vitro cell-based studies;	LAMP-2A mediated 5-FU induced histone deacetylation in CRC	5-FU promotes the degradation of p300/CBP via CMA autophagy, which is relevant to the chemoresistance of 5-FU [29].
LAMP-2A	HCT-116, DLD-1, NCM460 cell lines, in vitro cell-based studies	Upregulated LAMP-2A	High LAMP-2A expression is responsible for 5-FU resistance through the activation of the NF-κB pathway in CRC cell lines [106].
LAMP-2B	DLD1 cells, in vitro cell-based studies	Upregulated LAMP-2B	LAMP-2B silencing abolishes the fusion of autophagosomes with lysosomes and preserves cell viability in cells chronically exposed to resveratrol [74].
LAMP-3	CRC pan-cancer data omics	Upregulated LAMP-3	High LAMP3 is involved in the immune-associated processes and signaling pathways [94].
LAMP-3	CRC, xenograft tumor model, screening of clinical material; NCM46, LoVo, HCT-116 cells, in vitro cell-based studies	Upregulated LAMP-3	Downregulation of LAMP-3 expression induced by curcumin restrains tumor growth in mice [107].
LAMP-3	CRC, screening of clinical material	Higher LAMP-3 expression in CRC cells than in non-cancerous cells; depends on the stage of the disease	LAMP3 may promote cancer progression and metastasis and cause resistance to treatment [108].
LAMP-3	HCT116 cells, in vitro cell-based studies	Upregulated LAMP-3 as a novel TP53 target gene	LAMP-3 role in mediating the 5-FU-induced DNA damage response in TP53-proficient CRC cells [109].
LAMP-3	CRC, screening of clinical material;	Upregulated LAMP-3	High LAMP-3 expression has a poor overall survival [110].
LAMP-4 (CD68)	CRC, screening of clinical material; TCGA pan cancer data	Upregulated *CD68* (LAMP-4)	The expression levels of *CD68* correlate with the infiltration of immune cells in the pan-cancer microenvironment [95].
LAMP-5	CRC, TCGA and GEO repository	Upregulated LAMP-5	High expression correlates with shorter overall survival [111].
LAMP-5	CRC, GEO repository	Upregulated LAMP-5	Overexpression correlates with low survival [99].

## 6. Autophagy, LAMPs, and CRC Metastasis

Despite the progress made in early detection and targeted therapies for cancer over the last decade, CRC continues to be the second-leading cause of cancer-related deaths. At this stage, treatment typically includes chemotherapy combinations like oxaliplatin–irinotecan and 5-fluorouracil (5-FU) [112,113]. Adjuvant therapies can lead to drug resistance and disease progression, significantly impacting survival rates. Therefore, a considerable amount of work remains to be carried out in order to improve patient outcomes and reduce mortality rates [47]. In this regard, the complex molecular networks governing the distinct autophagic pathways in relation to carcinogenesis and particularly to CRC have been the focus of extensive research [114].

A reduction in essential autophagy proteins can hinder cancer growth, lower oxygen consumption, and lead to the buildup of dysfunctional mitochondria [115]. Notably, autophagy is also essential for the growth of RAS-driven tumors [116], including CRC [117].

Besides its critical role in regulating protein turnover and cancer immunogenicity, autophagy is involved in epithelial-to-mesenchymal transition (EMT), a crucial multistep mechanism needed by tumor cells to metastasize [117,118]. As cancer cells become more invasive, autophagy levels increase, enhancing cell motility and promoting a stem cell-like phenotype. Several studies have shown that the redistribution of lysosomes to the cell periphery, with LAMP-1 playing an essential role, is crucial for lysosomal exocytosis [119,120]. This process in turn remodels the extracellular matrix and activates EMT, contributing to the ability of tumor cells to intravasate, survive in circulation [121], and ultimately extravasate to secondary sites [122]. At these new locations, autophagy aids in maintaining tumor cells in a dormant state [123,124], promoting quiescence and survival. The speed at which tumor cells adapt to their new environments can influence the colonization process, potentially leading to micrometastasis or macrometastasis [122].

Emerging evidence suggests that autophagy enhances the survival of dormant [123,124] and circulating tumor cells [125,126], particularly in stem-like subpopulations. Furthermore, autophagy is shown to promote survival and controls the pluripotency of cancer stem cells (CSCs) within the TME, thereby contributing to invasion and treatment resistance [125,126].

To establish successfully distant colonies, metastatic tumor cells must overcome various challenges, with autophagy playing a crucial role in their response to cellular stress [127]. Indeed, multiple environmental stressors are known to enhance metastasis, such as hypoxia, as well as those experienced by circulating tumor cells, including nutrient deprivation [128] and detachment from the extracellular matrix (ECM) [129,130,131,132], inducing autophagic flux.

Autophagy is regulated through both transcriptional and post-translational mechanisms in response to nutrient signaling pathways [131,133,134,135]. However, it is not yet fully understood whether the augmented autophagy linked to the advancement of invasive tumors is a consequence of diminished nutrient availability in the TME or whether the regulation of autophagy by nutrient-sensing pathways becomes uncoupled during tumor growth.

Several studies have found a correlation between increased autophagy and metastasis [136]. For instance, enhanced punctate LC3B staining is observed in various malignances such as breast cancer [137] and melanoma [138], suggesting that autophagy may contribute to the growth and development of cancer cells [127].

High expression levels of LAMP-2A have been identified in gastric cancer [88]. Other research indicated an increased expression of LAMP-1 and LAMP-2 on the surfaces of highly metastatic cells, specifically those associated with metastatic CRC, which the authors correlated with tumor progression and the acquisition of metastatic characteristics. Further studies in recent years demonstrated dysregulated expression levels of other LAMPs, notably LAMP-3, LAMP-4, and LAMP-5. The upregulation of these proteins is associated with poor prognostic outcomes and is linked to the invasion and metastasis of CRC. Moreover, LAMP-3 overexpression has recently been connected to invasion and metastasis in CRC (Table 1). Within this context, the overexpression of LC3B is positively correlated with lymph node metastasis, a characteristic typically regarded as an unfavorable prognostic indicator. Interestingly, elevated LC3B is also associated with improved overall survival outcomes, indicating a complex relationship between this protein and the prognosis in CRC [139].

Although much has been accomplished in the fight against CRC, there is still considerable work to be done to decipher the link between autophagy and the metastatic potential of CRC cells [114]. However, the question of whether autophagy might be a useful target in clinical efforts to prevent the deadliest aspect of cancer, metastasis, remains open [136].

## 7. Autophagy, Lysosomes, and Drug Resistance

CRC is the most frequently diagnosed malignant tumor, known for its aggressive nature, which includes invasive growth and the potential for distant metastasis. Despite the established treatment regimens, the mortality rates associated with CRC continue to rise each year. This poor prognosis is attributed not only to the high percentage of cases diagnosed at later stages but also to the resistance that tumor cells develop against chemotherapy and radiotherapy.

Drug resistance is emerging as a significant clinical challenge, driven by both genetic and epigenetic mechanisms, including adaptive signaling events in tumor cells and their microenvironment. The two RAS signaling pathways closely linked to tumorigenesis are the MAP kinase pathway, which regulates cell proliferation, and the PI3K pathway, which oversees cell metabolism and survival. Additionally, the JAK/STAT signaling pathway, responsible for mediating cellular responses to cytokines and growth factors, plays a crucial role in regulating tumor proliferation, angiogenesis, and metastasis. Current chemotherapy regimens, including 5-fluorouracil (5-FU), irinotecan, oxaliplatin, and capecitabine, often fall short due to poor efficacy, side effects, disease recurrence, and chemotherapy failure [140,141]. Many patients receiving 5-FU-based treatments experience such resistance. A recent study by Yue et al. provided insights into STAT3-induced 5-FU resistance in CRC tissues and cell lines [142].

Autophagy has emerged as a significant regulator of chemoresistance in CRC. When CRC cells undergo chemotherapy treatment, autophagy is activated, which enhances cell survival and drug tolerance, enabling these cells to effectively evade treatment [113,143].

Lysosomes are believed to significantly contribute to the development of multidrug resistance in cancer therapy. Many chemotherapeutic agents accumulate in lysosomes due to a pH gradient, leading to their protonation and retention within the acidic environment of the lysosomal lumen [144,145]. Drugs such as cisplatin [146], sunitinib [147], doxorubicin [148], and vincristine [149] are particularly affected. Cancer cells may increase the expression of drug transporters in lysosomal membranes, actively pumping drugs into lysosomes [5,145]. Thus, it P-glycoprotein and the ABC transporter A3 can mediate drug efflux, reducing the availability of drugs to their intended targets and diminishing lysosomal function [150]. To counteract these effects, cancer cells promote lysosomal biogenesis, further enhancing drug sequestration and resistance. Additionally, lysosomes may translocate to the plasma membrane to facilitate drug secretion into the extracellular matrix, contributing to resistance—a mechanism observed in certain types of cancers. Despite extensive research on the role of lysosomes in drug resistance, some recent studies challenge this prevailing model [151].

As discussed above, autophagy plays a significant role in EMT. Several studies have demonstrated that the redistribution of lysosomes to the cell periphery, with LAMP-1 playing an essential role [120], is vital for lysosomal exocytosis. This process in turn remodels the extracellular matrix and activates EMT, which are critical steps for both malignant transformation and cancer progression [152]. Cancer stem cells (CSCs) are key players in tumor initiation and progression and contribute to therapy resistance by activating EMT and inflammatory pathways [115], crucial for CSC survival within the TME, enhancing their resistance to treatment. In CRC, the transcription factor SOX2 increases both EMT and ABCC2 gene expression, leading to chemotherapy resistance through β-catenin activation. Additionally, SOX2 can elevate BECLIN1 levels, thereby promoting autophagy and further contributing to chemoresistance [115].

The Beclin1 signaling pathway plays a critical role in regulating both autophagy and apoptosis, which significantly impacts the effectiveness of anticancer therapies. Although Beclin1 exhibits an antiapoptotic function in response to chemotherapy, the precise mechanisms underlying this role remain inadequately understood. Evidence suggests that Beclin1 has a substantial influence on cancer cell death, ultimately affecting outcomes for patients with CRC. Research by Park et al. indicated that BECLIN1 expression could serve as a predictor of the efficacy of cytotoxic chemotherapy in CRC [153].

There is an ongoing discussion about the impact of infections on therapy resistance, particularly in relation to autophagy in CRC [115]. While infections are acknowledged as factors contributing to the development of CRC, one significant element is inflammation, which can be modulated by interleukin 6 (IL-6). This cytokine promotes the phosphorylation of Beclin1, consequently facilitating the development of drug resistance [154]. Furthermore, changes in the epigenetic profile of CRC cells are linked to the emergence of chemoresistance [155].

Clinically, the treatment protocol for CRC patients often includes the administration of oxaliplatin and 5-FU, with the induction of autophagy through genomic and epigenetic modifications being associated with resistance to these agents [156,157]. Of note, these chemotherapeutics have been suggested to alter autophagy in different ways, at least in cell lines that are resistant to these drugs. Zitkute et al. showed that oxaliplatin resistance and lack of response to 5-FU can work through differential and even opposing modulation of ATG7, ATG12, and autophagic flux [158].

Even though data on irinotecan and autophagy are scarce, it has been demonstrated for this chemotherapeutic that it can elicit contrasting responses in autophagy depending on the genetics of the cell lines used. Thus, for stable microsatellite cells, this drug (and oxaliplatin) can in fact decrease autophagic vacuoles [159]. On the other hand, upon p53 deletion, John et al. found that irinotecan can increase autophagy and suggested a p53-dependent concomitant activation of autophagic cell death and apoptosis in their cell line model [160].

Recent studies have also underscored a relationship between CMA and resistance to antitumor therapies [14,27,161], with LAMP-2A being identified as a mediator of cisplatin resistance in CRC [56].

## 8. Lysosomes as Therapeutic Targets of Autophagy Drugs

Current research efforts are concentrating on the complexity of autophagy regulation and the development of drugs aimed at targeting autophagy in various diseases, including CRC. A major task of modern oncology is the search for novel approaches to overcome multidrug resistance, where lysosomes in particular have raised interest as potential pharmacological targets that can sensitize tumor cells to chemotherapy.

Many chemotherapeutic drugs are small lipophilic or amphiphilic molecules that accumulate inside lysosomes and passively diffuse across the lysosomal membrane into the lysosomal lumen [145]. The commonly used therapeutics cisplatin, sunitinib, doxorubicin, and vincristine are known to segregate in lysosomes [162]. Cancer cells can express drug transporters in the lysosomal membrane to pump cytotoxic drugs into the lysosome [5,145]. The ABC transporter P-glycoprotein, most commonly found on the plasma membrane, can also be expressed in the lysosomal membrane, where it mediates drug efflux into the lysosomal lumen. The ABC transporter A3 similarly induces lysosome-mediated multidrug resistance in leukemia cells [150].

Lysosomal cell death is dependent primarily on lysosomal membrane permeability and lysosomal acid proteases—cathepsin activity. Lysosomal membrane permeability is characterized by compromised lysosomal membrane integrity, which enables the leakage of lysosomal content into the cytoplasm [163]. There is a multitude of compounds with anticancer activity that induce changes in the lysosomal compartment of cancer cells. They include: lysosomotropic compounds and detergents, antihistamines, immunosuppressors, photosensitizers, thiosemicarbazone analogues, and plant derived substances. All of them accumulate in lysosomes of target cells and damage them by diverse mechanisms, as presented on Table 2.

### 8.1. Lysosomotropic Drugs

Basic players in strategies for sensitizing tumor cells to chemotherapy are the lysosomotropic compounds, which accumulate within the lysosomal lumen and induce cytoplasmic vacuolization, inhibition of lysosomal enzymes, and cell death. These molecules are readily stored in lysosomes of cancer cells, as their pH is much lower than that of normal cells [164].

Whilst lysosomes are overactivated in cancer cells, they are often more prone to lysosomal membrane permeabilization agents, which is another approach to induce cell death [165,166]. Studies on mouse embryonic fibroblasts demonstrated that *Bax/Bak1*-deficient cells treated with a lysosomotropic detergent showed increased lysosomal membrane permeability and restored autophagy [167]. Several authors reported that the increased permeability of lysosomal membranes can expand the transport of anticancer drugs [5,10]. It has been proven that the autophagy inhibitor chloroquine diphosphate improves the effect of 5-FU on colon cancer cells, suggesting that autophagy potentiates resistance to chemotherapy [168]. Sphingosine, an endogenous lysosomotropic agent, induces leakage of hydrolytic enzymes into the cytosol and triggers cell death. In Jurkat T-lymphoma cells and J774 cells treated with sphingosine at low to moderate concentrations, the partial rupture of lysosomes precedes caspase activation, while high concentrations of the same agent led to total rupture of lysosomes and result in necrosis without any preceding caspase activation or apoptosis [169]. Sphingosine also mediates the TNF-alpha-induced permeabilization of the lysosomal membrane, thus causing programmed cell death in various hepatoma cell lines [170]. The EMT inducer sphingosine-1-phosphate (S1P) mediates cancer stemness and 5-FU resistance, implicating it as a therapeutic target for CRC. Its inhibitor (ABC294640), combined with 5-FU, significantly suppresses tumor growth in mice and enhances 5-FU response in therapy-resistant CRC patient-derived tumor organoids [171].

An interesting observation is the unexpectedly strong antitumor effect of the antidepressant drug siramesine on breast, pancreas, nervous system, bladder, and lung cancer cells, which overexpress receptors binding this sigma-2 receptor antagonist. It acts as a lysosomotropic detergent and induces death of MCF-7 breast cancer cells by direct disruption of the lysosomal membrane and leakage of cathepsins into the cytosol [5]. Cathepsins are known to correlate with the metastatic capacity and aggressiveness of tumors. The decrease in LAMP-1 and LAMP-2 expression in transformed murine embryonic fibroblasts is cathepsin-dependent and sensitizes the cells to lysosomal cell death pathways induced by various anticancer drugs like cisplatin, etoposide, doxorubicin, and siramesine [172]. The cathepsin-dependent downregulation of LAMPs increases drug sensitivity in human colon and breast carcinoma cells, while the increase in LAMP levels by cathepsin inhibitors protects transformed cells against the lysosomal cell death pathway. Fehrenbacher et al. also showed that the knockdown of LAMP-1 or LAMP-2 is sufficient to sensitize the cells to siramesine-induced cell death and photooxidation-induced lysosomal destabilization, indicating that lysosome-targeting compounds are promising agents for cancer therapy [172].

Methylamine and siramesine, like chloroquine, leads to intracellular redistribution of cytostatics from the lysosomal lumen to the cytosol and have been suggested for combination therapies to overcome resistance [150]. Combining ammonium chloride or methylamine with vincristine and doxorubicin noticeably increases the response of resistant KBV1 cells to the chemotherapeutic treatment. Also, combining siramesine with vincristine has more pronounced effects than monotherapy in vitro and in vivo in breast cancer cells [149]. All these studies evidence the synergistic effect of cytostatic drugs and the suppressed lysosomal function in cancer.

### 8.2. Antihistamines with Anticancer Activity

Another group of lysosomotropic drugs are antihistamines like astemizole, loratadine, and ebastine, which accumulate more effectively in acidic tumors than in healthy tissues. Their use in combination with chemotherapeutics sensitizes cancer cells to chemotherapy and reverses multidrug resistance [173]. Loratadine inhibits the growth of tumors derived from human colon cancer cells (COLO 205) in vivo. In vitro studies demonstrated that this H1 receptor antagonist induced G2/M cell cycle arrest and apoptosis [174]. Another H1 receptor antagonist, meclizine, triggers apoptosis in human colon cancer cell lines (COLO 205 and HT 29 cells) by downregulating the Bcl-2 protein and by arresting cells in the G0/G1 phase [175]. It has been reported that enhanced H2 receptor gene expression is associated with prolonged survival in CRC patients [176]. Additionally, H1-antihistamines potentiate the activity of immunotherapy by enhancing T-cell activation in the TME [177]. Another H1 blocker, promethazine, has been reported to suppress the proliferation and to induce the apoptosis of CRC cells by inhibiting the PI3K/AKT signaling pathway [178]. A recent review highlighted the use of histamine H2 receptor (H2R) ligands in cancer with two main adjuvant applications: improving antitumor efficacy by regulation of immune response, and preventing toxic adverse effects produced by chemo- or radiotherapy [179]. All these data suggest that the accumulation of antihistamines in lysosomes induces cancer cell death and may be a reliable option as a complementary therapy in CRC.

### 8.3. Photosensitizer Drugs

Photosensitizers are also implicated in lysosomal cell death. Novel porphyrin and phthalocyanine photosensitizers are designed for targeted photodynamic therapy of CRC. Jans et al. showed that the Pc9-T1107 photosensitizer is localized primarily in lysosomes and endoplasmic reticulum of CT26 cells, as these organelles are the initial sites of photodamage [180]. The ROS generated by the photodynamic process cause rapid damage of lysosomes, leading to the release of cathepsins into the cytosol. The activation of the Bax protein in turn initiates the mitochondrial apoptosis pathway [181]. A recent study by Fu et al. reported the effects of C_60_ nano-photosensitive drugs conjugated with 5-FU in CRC treatment. Photosensitized tumor-targeted drug delivery led to apoptosis and showed higher antitumor efficacy and safety in vitro and in vivo on CRC samples compared to free 5-FU, with no significant toxic effects on normal cells or tissues [182].

### 8.4. Thiosemicarbazone Analogues

Thiosemicarbazone analogues, iron-chelating compounds with strong antiproliferative effects, are known to activate selectively the lysosomal pathway. They generate oxidative stress in cancer cells, permeabilize the lysosomal membrane, and increase cytotoxicity, which contribute to overcoming resistance to anticancer drugs [183]. Thiosemicarbazones can also activate apoptosis and autophagy and inhibit c-Met oncogene expression through lysosomal degradation [184]. Knowing the implication of iron in the pathophysiology of CRC, Akam and Tomat examined the biological activity of prochelators in CRC cells and proved that glycoconjugate prochelation potentiates the preferential uptake by cells overexpressing glucose transporters [185]. An original approach was proposed by Kaya et al., who designed novel N-acridine thiosemicarbazones targeting lysosomes in several caner types, including HCT-15 colon adenocarcinoma cells. The authors considered this strategy useful in overcoming Pgp-mediated resistance, which is the death-causative agent in advanced and resistant cancer [186].

### 8.5. Other Modulators of Autophagy

Autophagy-modulating agents have been proposed as chemosensitizers for cisplatin therapy in cancer [187]. The Warburg effect, typical of cancer cells, enables them to use glycolysis as a primary source of ATP. The alerted ATP–ADP ratio leads to changes in autophagy and cell death, which may enhance the antitumor efficacy of many chemotherapeutics. The glycolysis inhibitor 2-deoxy-D-glucose (2-DG) is a proven chemosensitizer that induces apoptosis in RKO colon carcinoma cells. It also reduces apoptosis in the HCT116 colon carcinoma cell line, suggesting a cell line-specific effect. In contrast, 2-DG releases HCT116 from cisplatin-induced apoptosis, which is even more pronounced after the addition of rapamycin [188].

Andrographolide is a naturally occurring labdane diterpenoid with anti-inflammatory, antiviral, antioxidant, and anticancer activity. It activates Nrf2, which in turn facilitates oxidative stress response, thus limiting ROS accumulation. It also sensitizes colon cancer cell lines in vitro towards cisplatin-induced apoptosis [189].

Epigallocatechin gallate (EGCG) is a polyphenolic catechin present in green tea with strong anticancer activity. It acts synergistically with cisplatin or oxaliplatin, reducing cell proliferation and causing cell death in colorectal cancer cell lines. This effect is associated with increased autophagosome formation and accumulation [190]. The same team showed that siRNA knockdown of autophagy-related ATG genes reversed the EGCG chemosensitizing effect, which suggests the involvement of autophagic cell death. Another key chemotherapeutic, especially in metastatic CRC, is irinotecan, and this drug has also been shown to trigger autophagic cell death in gastric cancer cells [191]. In line with the abovementioned modes of action of EGCG, Wu et al. demonstrated that this catechin synergizes with irinotecan as well to enhance DNA damage and decrease migration and invasion in RKO and HCT116 cells. Importantly, these effects were concomitant with increased autophagosome formation measured by LC3B-II-to-LC3B-I transformation [192].

S-adenosyl-L-methionine (AdoMet) is a sulfur-containing nucleoside with anticancer potential. Recently, Mosca et al. demonstrated in a p53 knockout model of HCT116 cells that were also depleted of the ribosomal protein uL3 that this nucleoside can effectively inhibit the formation of autophagolysosomes (autophagic flux) and can decrease the protein levels of Atg7 and the LC3B-II-to-LC3B-I ratio, indicating perturbed macroautophagy. Importantly, AdoMet resensitized cells to 5-FU [193].

Interestingly, 3-methyladenine (3-MA) has been successfully used in vitro to reduce cell growth and malignant phenotypes (as assessed by colony formation assays) of CRC cells that survive a double combination of 5-FU and oxaliplatin. Baldasso-Zanon et al. demonstrated that these chemotherapeutics induce cytoprotective autophagy, which peaks at day 3–5 after treatment, and inhibition with 3-MA within this time window (not simultaneously with the administration of the two drugs) may present a potential therapeutic strategy in CRC [194]. Nevertheless, as Shi et al. had previously reported, the inhibition of autophagy with 3-MA (or knockdown of ATG5 and Beclin1) can make Caco-2 cells more sensitive to oxaliplatin therapy, leading to enhanced cell death, so targeting these cytoprotective mechanisms at any point during therapy may be beneficial [195].

Lithium is another agent known to induce autophagy. CRC cells treated with lithium present with ineffective induction of autophagy, which is suspended at late stages. It chemosensitizes cancer cells towards cisplatin or 5-FU. Additionally, lithium induces depletion of LAMP-1, LAMP-2, and cathepsin B, suggesting comprised lysosomal stability. Combined with oxaliplatin, it synergistically inhibited tumor growth in a xenograft colorectal carcinoma model [196].

**Figure 2:**
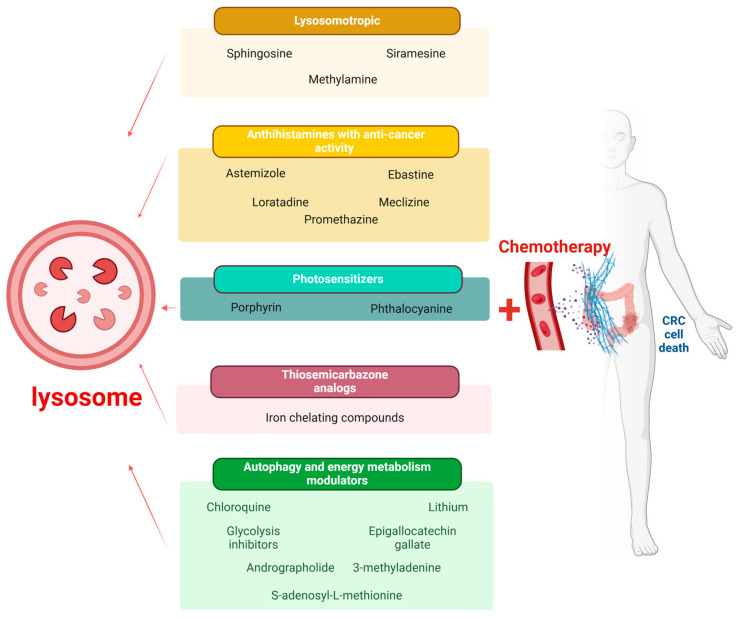
Lysosomes as therapeutic targets of autophagy drugs. The figure represents a multitude of compounds with anticancer activity that induce changes in the lysosomal compartment of cancer cells. All of them accumulate in lysosomes of the target cells and damage them by diverse mechanisms. That in turn promotes the efficiency of chemotherapy treatment by enhancing drug sensitivity and inducing cancer cell death.

In summary, lysosomal proteins not only favor resistance by drug sequestration being the “final destination” for cytostatic drugs but also act as a “signaling hub”, activating cell survival pathways [5]. As far as LAMPs are concerned, there is evidence for their direct involvement in drug resistance. A study by Guo et al. showed that LAMP-1 is upregulated in breast cancer cells upon development of resistance by continuous exposure to doxorubicin [197]. There are also several investigations revealing upregulation of LAMPs in different malignancies. LAMPs are overexpressed in tumor cells of basocellular carcinomas [81]. The same authors detected elevated LAMP-1 at the protein and transcriptional level in glioblastoma [198]. LAMP-2, considered the main regulator of CMA, is detected with the highest transcriptional activity in clinical stage II of CRC [153]. This finding indicates that the inhibition of CMA may be an effective treatment for highly advanced stages of cancer. Inhibition of LAMP-2 reverses macrophage activation, increases tumor cytotoxicity, and inhibits cancer progression [199]. Cisplatin may affect lysosomal stability both directly (by changing membrane fluidity) and indirectly by increasing ROS production and decreasing LAMP-1 and LAMP-2 expression. Furthermore, cisplatin-resistant cancer cells are characterized by reduction in the lysosomal compartment with downregulation of LAMP-1 and LAMP-2 [200]. Figure 2 illustrates the implication of lysosomes as therapeutic targets of autophagy drugs.

**Table 2:** An overview of lysosomes as therapeutic targets in human CRC.

Drug Classifications	Name of Drug	Mechanism of Action
**Lysosomotropic**	**Chloroquine**	Accumulates within the lysosomal lumen and induces cytoplasmic vacuolization, inhibition of lysosomal enzymes and cell death. These molecules are readily stored in lysosomes of cancer cells as their pH is much lower than that of normal cells [168].
	**Sphingosine**	Induces leakage of hydrolytic enzymes into the cytosol and triggers cell death [169]. Mediates TNF-alpha induced permeabilization of the lysosomal membrane, thus causing programmed cell death [170].
	**Siramesine**	Disrupts the lysosomal membrane, inducing a leakage of cathepsins into the cytosol [5]. Decreases LAMP-1 and LAMP-2 expression [172].
	**Methylamine**	Leads to intracellular redistribution of cytostatics from the lysosomal lumen to the cytosol [149,150].
**Antihistamines with anticancer activity**	**Astemizole**	Sensitizes cancer cells to chemotherapy and reverses multidrug resistance [173].
	**Loratadine**	Induces G2/M cell cycle arrest and apoptosis [174].
	**Ebastine**	Potentiates the activity of immunotherapy by enhancing T cell activation in the TME [177].
	**Meclizine**	Triggers apoptosis in human colon cancer cell lines (COLO 205 and HT 29 cells) by downregulating the Bcl-2 protein and by arresting cells in the G0/G1 phase [175].
	**Promethazine**	Suppresses the proliferation and induces apoptosis of CRC cells by inhibiting the PI3K/AKT signaling pathway [178].
**Photosensitizers**	**Porphyrin**	The photodynamic process generates ROS and causes rapid damage of lysosomes, leading to the release of cathepsins into the cytosol. Activation of the Bax protein initiates mitochondrial apoptosis pathway [181].
	**Phthalocyanine**	C60 nano-photosensitive drugs conjugated with 5-FU in CRC treatment induce oxidative stress [182].
**Thiosemicarbazones**	**Iron chelating compounds**	Generate oxidative stress in cancer cells, permeabilize the lysosomal membrane, and increase cytotoxicity, which contribute to overcoming resistance to anticancer drugs [183]. Activate apoptosis and autophagy and inhibit c-Met oncogene expression through lysosomal degradation [184,185,186].
**Autophagy and energy metabolism modulators**	**Andrographolide**	Activates Nrf2, which facilitates oxidative stress response, thus limiting ROS accumulation. Sensitizes CRC cell lines in vitro towards cisplatin-induced apoptosis [189].
	**Epigallocatechin gallate (EGCG)**	Increases autophagosome formation and accumulation [190]. Acts synergistically with cisplatin or oxaliplatin, reduces cell proliferation, and causes cell death in colorectal cancer cell lines. Synergizes with irinotecan to enhance DNA damage and decreases migration and invasion [191]. Increases autophagosome formation as measured by LC3B transformation [192].
	**S-adenosyl-L-methionine (AdoMet)**	Inhibits the formation of autophagolysosomes and decreases the protein levels of Atg7 and the LC3B-II to LC3B-I ratio. Resensitizes cells to 5-FU [193].
	**3-methyladenine (3-MA)**	Reduces the cell growth and the malignant phenotype of CRC cells that survive a double combination of 5-FU and oxaliplatin [194]. Enhances cell death of Caco-2 cells by inhibiting autophagy [195].
	**Lithium**	Ineffective induction of autophagy, which is suspended at late stages. Chemosensitizes cancer cells towards cisplatin or 5-FU. Induces depletion of LAMP-1, LAMP-2 and cathepsin B. Inhibits tumor growth in a xenograft colorectal carcinoma model with oxaliplatin [196].

## 9. Conclusions

Despite the numerous efforts to improve chemotherapy, treatment failure and resistance mechanisms remain a major challenge in CRC. Autophagy has been strongly implicated in tumorigenesis and drug response, and several groups of compounds such as lysosomotropic agents, thiosemicarbazone, and nucleotide/nucleoside analogues have been demonstrated to successfully modulate autophagy and enhance (either additively or synergistically) the cytotoxic effects of 5-FU, oxaliplatin, and irinotecan, at least in vitro. The involvement of lysosomes in drug resistance opens a promising research field aiming to overcome chemoresistance. New approaches may target not only lysosomal mechanisms implicated in autophagy but also lysosome-associated signaling pathways and lysosome-restricted proteins like LAMPs. Interfering with lysosomal function and affecting LAMPs might be a promising approach enabling sensitization of CRC cells to chemotherapy. Although hampering lysosomal function may affect not only tumor cells but a multitude of normal cells as well, lysosomal proteins and LAMPs especially should be in the spotlight of future research. These molecules may serve as specific targets in drug-resistant cancer cells, enabling an increase in the therapeutic selectivity of anticancer agents.

## Data Availability

The data that support the review are available from the corresponding authors upon reasonable request. Contact Tsvetomira Ivanova or Victoria Sarafian.
